# 免疫检查点抑制剂相关皮肤不良反应诊治建议

**DOI:** 10.3779/j.issn.1009-3419.2019.10.06

**Published:** 2019-10-20

**Authors:** 晓燕 斯, 春霞 何, 丽 张, 小伟 刘, 玥 李, 汉萍 王, 潇潇 郭, 佳鑫 周, 炼 段, 力 张

**Affiliations:** 1 100730 北京，中国医学科学院，北京协和医院呼吸与危重症医学科 Department of Pulmonary and Critical Care Medicine, Peking Union Medical College Hospital, Beijing 100730, China; 2 100730 北京，中国医学科学院，北京协和医院皮肤科 Department of Dermatology, Peking Union Medical College Hospital, Beijing 100730, China; 3 100730 北京，中国医学科学院，北京协和医院检验科 Department of Clinical Laboratory, Peking Union Medical College Hospital, Beijing 100730, China; 4 100730 北京，中国医学科学院，北京协和医院眼科 Department of Ophthalmology, Peking Union Medical College Hospital, Beijing 100730, China; 5 100730 北京，中国医学科学院，北京协和医院消化内科 Department of Gastroenterology, Peking Union Medical College Hospital, Beijing 100730, China; 6 100730 北京，中国医学科学院，北京协和医院心内科 Department of Cardiology, Peking Union Medical College Hospital, Beijing 100730, China; 7 100730 北京，中国医学科学院，北京协和医院风湿免疫科 Department of Rheumatology, Peking Union Medical College Hospital, Beijing 100730, China; 8 100730 北京，中国医学科学院，北京协和医院内分泌科 Department of Endocrinology, Peking Union Medical College Hospital, Beijing 100730, China

**Keywords:** 免疫检查点抑制剂, 免疫相关不良反应, 皮肤不良反应, Immune checkpoint inhibitor, Immunotherapy-related toxicities, Dermatologic toxicities

## Abstract

免疫检查点抑制剂已应用于多种恶性肿瘤的治疗。免疫检查点抑制剂会引起免疫相关不良反应，其中皮肤不良反应是常见的不良反应之一。皮肤不良反应最常表现为斑丘疹和瘙痒。严重的皮肤不良反应包括Stevens-Johnson综合征、中毒性表皮坏死松解症等。本文综合各个免疫治疗相关不良反应管理指南、皮肤不良反应个案报道及综述，并结合临床实践，提出了免疫相关皮肤不良反应的诊治建议。

## Abstract (en)

Immune checkpoint inhibitors (ICIs) represent a major breakthrough in cancer therapy. Immune-related adverse events (irAEs) may occur during treatment due to their unique mechanism of action. Dermatologic toxicities appear to be one of the most prevalent irAEs. The most common symptoms are maculopapular rash and pruritus. Serious dermatologic AEs, including Stevens-Johnson syndrome and toxic epidermal necrolysis, are rare. In this review, we summarized guidelines of management of immunotherapy-related toxicities and case reports, and proposed treatment recommendation.

## 1 概况

免疫检查点抑制剂（immune checkpoint inhibitors, ICIs）已成功应用于多种恶性肿瘤的治疗，如黑色素瘤、非小细胞肺癌、肾细胞癌、霍奇金淋巴瘤等。常见的ICIs包括细胞毒T淋巴细胞相关抗原-4（cytotoxic T lymphocyte-associated antigen-4, CTLA-4）单克隆抗体ipilimumab、程序化细胞死亡蛋白（programmed death 1, PD-1）单克隆抗体纳武利尤单抗（nivolumab）、帕博利珠单抗（pembrolizumab）及程序化细胞死亡配体-1（programmed death ligand 1, PD-L1）单克隆抗体atezolizumab。在免疫检查点抑制剂使用过程中会发生免疫相关不良反应（immune-related adverse events, irAEs）。皮肤不良事件是最常见的irAEs，发生率分别为ipilimumab 43%-45%，纳武利尤单抗和帕博利珠单抗34%^[1]^。皮肤毒性可以在治疗早期出现（治疗开始后的前几个星期），也有报道在治疗结束后出现皮肤不良反应的病例^[2]^。

免疫相关皮肤不良反应的发生机制目前尚不完全明确，但显然是和PD-1/PD-L1和CTLA-4受体阻断介导的T细胞活化相关^[3]^。文献^[4]^报道了73例晚期非小细胞肺癌患者接受纳武利尤单抗或帕博利珠单抗，25例（34.2%）出现免疫相关皮肤不良反应，疗效为完全缓解/部分缓解的患者比疗效为疾病稳定/疾病进展的患者皮肤不良反应发生率高（68.2% *vs* 19.6%）。肿瘤组织和皮肤活检组织中有9种共同抗原，在体外能激活CD4^+^和CD8^+^ T细胞。

免疫相关皮肤不良反应多数较轻，严重的不良反应较为罕见。目前报道的有伴嗜酸性粒细胞增多和系统症状的药疹（drug rash with eosinophilia and systemic symptoms, DRESS）、急性发热性嗜中性皮病（acute febrile neutrophilic dermatosis, Sweet综合征）、Stevens-Johnson综合征或中毒性表皮坏死松解症（toxic epidermal necrolysis, TEN）。

大多数免疫相关皮肤不良反应对治疗有反应，而生物制剂对皮质类固醇难治性疾病患者有效。嗜酸性粒细胞增多、白介素-6（interleukin 6, IL-6）、白介素-10（interleukin 10, IL-10）、免疫球蛋白E（immunoglobulin E, IgE）升高与免疫相关皮肤不良反应相关，可能是皮肤不良反应治疗的靶点^[5]^。

## 2 各种皮肤不良反应处理

美国国立综合癌症网络（National Comprehensive Cancer Network, NCCN）、欧洲肿瘤内科学会（European Society for Medical Oncology, ESMO）和中国临床肿瘤协会（Chinese Society of Clinical Oncology, CSCO）都有免疫治疗相关不良反应管理的临床指南。在使用ICIs之前做基线皮肤检查，已知有免疫相关性皮肤病史的患者应该到皮肤科就诊进行基线皮肤检查，如大疱性类天疱疮、银屑病、扁平苔藓、红斑狼疮。发生皮肤不良反应时应详细询问病史，包括皮疹严重程度、合并症、伴随用药；查体需要包括全身皮肤检查，皮疹形态（特别是水疱）、皮疹的面积，黏膜是否受累。确诊免疫相关皮肤不良反应时，需除外其他皮肤问题，如感染、其他药物作用、其他系统性疾病、原发皮肤疾病。

### 2.1 斑丘疹

斑丘疹是免疫治疗引起的最常见的皮疹。NCCN免疫相关毒性反应管理指南（2019年第1版）将斑丘疹的严重程度分为轻度（1级）、中度（2级）和重度（3级-4级）。CSCO免疫检查点抑制剂相关的毒性管理指南按照常见不良事件评价标准（Common Terminology Criteria for Adverse Events, CTCAE4.0）将斑丘疹分为3级。ESMO免疫治疗的毒性指南将皮疹分为4级^[1]^。ESMO指南提出，当皮疹面积定义30%时，皮疹分级为3级是否合适。当皮疹尽管弥漫但程度较轻时，分为2级更合适。三个指南总体的处理推荐都是一致的（表 1）。对于2级的皮疹，ESMO指南推荐继续使用ICIs，而NCCN指南和CSCO指南建议可以考虑暂停ICIs。因此，对于弥漫但程度较轻的皮疹，ESMO指南更倾向于继续使用ICIs。NCCN指南对于系统使用糖皮质激素的患者，在症状改善至≤1级后，4周-6周逐步减量停药，推荐的治疗疗程比ESMO指南稍长。

**1:** 免疫治疗相关斑丘疹管理 Management of immunotherapy-related maculopapular rash

Grades		ESMO guidelines	NCCN guidelines	CSCO guidelines
1	Macules/papules covering < 10% BSA with or without symptoms (*e.g*., pruritus, burning, tightness)	Continue immunotherapy Avoid skin irritants and sun exposure Topical emollients Topical steroids (mild strength) cream Oral or topical antihistamines for itch	Continue immunotherapy Topical emollient Oral antihistamine Treatment with moderate potency topical steroids to affected areas	
2	Macules/papules covering 10%-30% BSA with or without symptoms (*e.g*., pruritus, burning, tightness); limiting instrumental activities of daily living	Continue immunotherapy Supportive management as above, topical steroids (moderate or potent strength) cream	Consider holding immunotherapy Topical emollient Treatment with high potency topical steroids to affected areas AND/OR Prednisone 0.5 mg/kg/d-1 mg/kg/d	
3	Macules/papules covering > 30% BSA with or without associated symptoms; limiting self-care activities of daily living	Withhold immunotherapy Topical treatments as above (potent) If mild or moderate oral 0.5 mg/kg-1 mg/kg prednisolone If severe *i.v*. (methyl) prednisolone	Hold immunotherapy Treatment with high potency topical steroids to affected areas Prednisone 0.5 mg/kg/d-1 mg/kg/d (increase dose up to 2 mg/kg/d if no improvement) Urgent dermatology consultation Consider inpatient care	
4	Skin sloughing > 30% BSA associated symptoms (*e.g*., erythema, purpura, epidermal detachment)	Discontinue immunotherapy *i.v*. (methyl) prednisolone 1 mg/kg-2 mg/kg Seek urgent dermatology		
ESMO: European Society for Medical Oncology; NCCN: National Comprehensive Cancer Network; CSCO: Chinese Society of Clinical Oncology; BSA: body surface area.				

需要注意的是，斑丘疹可能是其他免疫相关皮肤不良反应的早期表现，如苔藓样反应、银屑病、大疱性类天疱疮等。因此对于不典型的、严重的、持续反复的皮疹，需要行进一步的检查，特别是皮肤活检。

### 2.2 瘙痒

瘙痒也是ICIs引起的最常见的不良反应之一。荟萃分析显示纳武利尤单抗和帕博利珠单抗引起的所有级别的瘙痒发生率为13%-20%^[6]^。在ipilimumab和联合使用时发生率更高，在抗PD-L1单抗的治疗中发生率略低。瘙痒可以与皮疹同时存在，也可以出现在皮肤外观正常的患者中。NCCN指南和CSCO指南将瘙痒分为3级（表 2），两个指南对于瘙痒的处理基本一致。文献报道^[7]^阿瑞匹坦（80 mg，每日1次，服用5 d）治疗纳武利尤单抗引起的难治性瘙痒。

**2:** 免疫治疗相关的皮肤瘙痒管理 Management of immunotherapy-related pruritus

Grades		NCCN guidelines/CSCO guidelines
1	Mild or localized	Continue immunotherapy Oral antihistamines Treatment with moderate potency topical steroids to affected areas
2	Intense or widespread; intermittent; skin changes from scratching (*e.g*., Edema, papulation, excoriations, lichenification, oozing/crusts); limiting activities of daily living.	Continue immunotherapy with intensified antipruritic therapy Oral antihistamines Treatment with high potency topical steroids to affected areas Dermatology consultation
3	Intense or widespread; constant; limiting self-care activities of daily living or sleep.	Hold immunotherapy Oral antihistamines Prednisone/methylprednisolone 0.5 mg/kg/d-1 mg/kg/d Consider GABA agonists (gabapentin, pregabalin) Consider aprepitant or omalizumab (for increased IgE) for refractory cases Urgent dermatology consultation

### 2.3 苔藓样皮炎

苔藓样皮炎可在治疗后数周至数月后出现。临床上可表现为脓疱、丘疹和斑块。病理上可表现为带状淋巴细胞浸润、角化过度、颗粒层和棘层增厚、角化不良，可伴有明显的表皮增生；也可表现为角化不全、海绵状水肿、皮肤附属器/血管周围炎症和嗜酸性粒细胞浸润。治疗主要是局部外用糖皮质激素，少数情况需要口服糖皮质激素、光疗和阿维A。

### 2.4 银屑病

在ICIs治疗过程中可出现银屑病病情加重或是新发银屑病，新发的银屑病常在用药数月后出现，掌跖和头皮都可能受累，可伴有银屑病关节炎。常见斑块型银屑病，可同时出现点滴型银屑病、掌跖银屑病或掌跖脓疱病。ICIs引起银屑病的病理机制尚未明确。可能与PD-1轴下调T辅助细胞1（Th1）/Th17信号通路有关。PD-1抑制可以促进Th17淋巴细胞介导的促炎细胞因子过度表达。这些患者还可能有血清IL-6水平的升高^[8]^。治疗上，可以局部使用糖皮质激素、光疗、口服阿维A、全身应用糖皮质激素。文献报告了1例帕博利珠单抗诱发银屑病的晚期黑色素瘤患者，对全身性皮质类固醇治疗效果不佳，使用白介素-17A（interleukin 17A, IL-17A）抑制剂苏金单抗（secukinumab）后银屑病缓解^[9]^。目前认为抗TNF-α治疗可能效果不佳。对伴有银屑病关节炎的患者，可以考虑使用甲氨蝶呤、全身糖皮质激素。

### 2.5 白癜风

白癜风多数发生在使用ICIs的黑色素瘤患者中，但偶尔也有其他肿瘤的报道^[10, 11]^。荟萃分析显示帕博利珠单抗和纳武利尤单抗使用过程中发生率分别为8.3%和7.5%^[6]^。免疫治疗相关的白癜风可能与黑色素瘤细胞和正常黑色素细胞共有的抗原交叉反应相关。白癜风多数发生在治疗后的数月，常见的为双侧对称分布。白癜风样皮疹在治疗停止后仍会持续。在黑色素瘤治疗中，白癜风可能是疗效好的预测因子。有个案报道，白癜风的再色素化与黑色素瘤复发相关^[12]^。

### 2.6 大疱性类天疱疮（bullous pemphigoid, BP）

ICIs治疗可引起免疫相关的BP，或者可以使之前存在的BP加重。BP可在ICIs治疗后快速发生，也可在治疗数月后发生。前期可表现为瘙痒和非特异性斑丘疹，黏膜受累少见。直接免疫荧光显示基底膜带IgG和补体C3的线状沉积。抗BP230抗体可为阳性。出现BP的患者需要停用ICIs。治疗上可使用局部或全身皮质类固醇。也有文献报道使用利妥昔单抗和奥马珠单抗。有报道在停止使用ICIs数月后仍有皮疹的持续。Lopez等^[13]^总结了21例ICIs相关的BP患者，多数发生在用药后6个月-8个月内，在BP出现之前或出现时多数伴有明显的瘙痒，大多数患者（76%）需要停用ICIs。

### 2.7 皮肤毛细血管增生症（cutaneous capillary endothelial proliferation, CCEP）

王峰等^[14]^报道在卡瑞利珠单抗（camrelizumab）治疗原发性肝癌中出现CCEP，单药使用发病率为77.1%，多见于颜面部和体表皮肤，未见发生于呼吸道和消化道黏膜的病例。卡瑞利珠单抗单药引发CCEP患者客观有效率较高。卡瑞利珠单抗与阿帕替尼或FOLFOX4化疗方案（奥沙利铂、亚叶酸钙、氟尿嘧啶）联合使用能够降低CCEP发生率。CCEP的病理可表现为真皮层内薄壁血管呈簇状增生，血管充血扩张，见灶性伴血栓形成趋势。处理上主要以局部治疗防治感染，必要时可考虑激光、外科切除；伴有感染时暂停ICIs，并行抗感染治疗。

### 2.8 Stevens-Johnson综合征

Stevens-Johnson综合征是重症多形红斑型药疹。临床上表现为非特异性斑丘疹、水疱、表皮坏死剥脱，主要累及躯干，可出现黏膜和生殖器溃疡。可伴有发热、咽痛、关节痛或腹痛。病理显示为角质形成细胞凋亡，真皮淋巴细胞浸润和表皮分离，真表皮交界处及病灶处皮下有CD8^+^ T细胞浸润及坏死角质形成细胞。免疫组化：浸润的T细胞为CD8^+^ T细胞且表达PD-1。同时在T细胞浸润部位的皮肤角质形成细胞表达有PD-L1。出现Stevens-Johnson综合征需要永久停用ICIs，并请皮肤科急会诊，予泼尼松/甲基强的松龙1 mg/kg/d-2 mg/kg/d，静脉免疫球蛋白治疗。

Salati等^[15]^报道了1例女性肺鳞癌患者在使用nivolumab 3周后出现发热、乏力、痛性口腔溃疡，口唇出血结痂，下肢及掌跖斑丘疹。血液检查发现C反应蛋白升高，转氨酶升高。予甲基泼尼松龙1 mg/kg及营养支持治疗后，患者症状逐步缓解。Haratake等^[16]^报道了1例肺腺癌患者在使用帕博利珠单抗12天后出现发热、乏力，伴皮肤水痘样皮疹、口腔溃疡和结膜充血水肿。皮肤病理提示角质形成细胞坏死、棘层松解性大疱。治疗上采用泼尼松龙口服、地塞米松、左氧氟沙星滴眼。30天左右症状逐步缓解。

### 2.9 TEN

临床表现为广泛的红斑、水疱、大疱、表皮坏死、松解、剥脱，表皮松解或剥脱的面积≥全身体表面积的30%，伴有全身中毒症状和黏膜受累。病理表现为界面皮炎，真表皮交界处T淋巴细胞浸润和角质形成细胞凋亡以及表皮坏死伴少量T淋巴细胞浸润。免疫组化显示少量散在的淋巴细胞PD-L1染色阳性，表皮极少表达，主要集中在基底层角质形成细胞和黑色素细胞。治疗上需采用糖皮质激素和静脉免疫球蛋白。中毒性表皮坏死松解症死亡率高。

Vivar等^[17]^报道了1例女性黑色素瘤患者在使用ipilimumab 3 mg/kg和纳武利尤单抗注射液1 mg/kg后，出现麻疹样红斑、瘙痒，停用了ipilimumab，继续使用2周期纳武利尤单抗（3 mg/kg），3周期后皮疹加重。皮肤活检病理提示最初为界面皮炎，随后为表皮全层坏死。予泼尼松1 mg/kg、英夫利昔单抗、静脉免疫球蛋白治疗。但患者最终因合并感染死亡。

### 2.10 药疹伴嗜酸性粒细胞增多和系统症状（drug reaction with eosinophilia and systemic symptoms, DRESS）

DRESS表现为泛发性红斑，嗜酸粒细胞增多，很多累及肝脏，出现转氨酶升高。治疗上主要采用全身糖皮质激素。

Mirza等^[18]^报道了1例男性黑色素瘤患者，在接受ipilimumab和纳武利尤单抗后出现发热、头痛、面部、背部和胸部的斑丘疹。血常规提示嗜酸性粒细胞1.41×10^9^/L。血生化提示谷丙转氨酶（alanine aminotransferase, ALT）126 U/L，谷草转氨酶（aspartate aminotransferase, AST）116 U/L，肌酐（creatinine, Cr）1.2 mg/dL。治疗上给予甲基强的松龙100 mg静脉注射每日1次，2 d后皮疹、转氨酶好转。续贯口服泼尼松100 mg每日1次，每4天减10 mg，疗程共8周。

### 2.11 Sweet综合征

Sweet综合征又称为急性发热性嗜中性皮病（acute febrile neutrophilic dermatosis）。1964年由Robert Douglas Sweet提出。药物相关的Sweet综合征诊断主要依据以下临床特点：突然出现的痛性红斑或结节；组织病理为密集的中性粒细胞浸润但无白细胞破碎性血管炎；发热 > 38 ℃；药物暴露和临床表现之间的关系；停药或全身激素治疗后缓解。

Adler等^[19]^报道了1例男性黑色素瘤患者，使用4次ipilimumab后出现左手多个压痛性的紫红色结节，伴有全身不适、发热。血常规提示白细胞升高。皮肤活检病理提示真皮密集的中性粒细胞浸润伴水肿，无白细胞破碎性血管炎。停用ipilimumab，并予口服泼尼松1 mg/kg/d，1个月内逐步减停；局部皮质激素软膏。患者皮疹完全恢复。

## 3 再次使用ICIs

对于斑丘疹或者瘙痒，在症状恢复至≤1级时，可以考虑再次使用ICIs。但是对于出现严重的威胁生命的水疱性疾病，如Stevens-Johnson综合征、中毒性表皮坏死松解症和DRESS，永久停用ICIs。

## 4 皮肤不良反应与疗效的关系

在接受抗PD-1单抗的黑色素瘤患者中，免疫相关的皮肤不良反应——白癜风与较好的临床疗效相关。

## 5 总结

免疫相关的皮肤不良反应虽然发生率高，但多数为轻度的皮肤不良反应，只有极少数会危及生命。加强患者教育，早期识别、处理严重皮肤不良反应，使ICIs治疗更加安全。
